# Lessons From Whakapapa and Filial Piety: Can Social Work Capitalize on the Connection That Survives Death?

**DOI:** 10.1093/geroni/igaa057.1351

**Published:** 2020-12-16

**Authors:** Hong-Jae Park

**Affiliations:** Western Sydney University, Penrith, Australia

## Abstract

Ageing is part of life, and so is death. Although death will involve all of us over time, it is often regarded as a taboo topic, and bonds with the dead are seldom acknowledged in contemporary times. The paper presents selected insights on the connection that survives death, learned from a qualitative study on two indigenous knowledges—whakapapa (genealogical connections in Maori) and filial piety (respect/care for ancestors). Data were collected from interviews with 49 key informants (Maori=25; Korean=24) in 2018/19 in New Zealand and South Korea. The research findings indicate that the connectedness with ancestors or deceased loved ones is a significant part of the participants’ mental and social lives. Māori (the first nation people of New Zealand) have established the unwritten convention of whakapapa as the core value that places whānau (family) at the centre of social relationships. In Korean culture, its filial piety/ancestor veneration tradition has emphasised the connection between deceased and living family members. Criticism about the traditions of whakapapa and filial piety was also raised by a few participants. The significance of this study is situated in the innovative perspective that the post-mortem relationship can be embodied, not only by the living who practise memorial respect for the dead, but also by those older people who establish after-life legacy before death. To help capitalise on this whakapapa connection, the so-called concept of “memorial social work” is presented as a potential area of social work practice, which has critical implications in the ageing/end-of-life related fields.

